# Characterization of QTLs for Root Traits of Wheat Grown under Different Nitrogen and Phosphorus Supply Levels

**DOI:** 10.3389/fpls.2017.02096

**Published:** 2017-12-11

**Authors:** Yongzhe Ren, Yingying Qian, Yanhua Xu, ChunQin Zou, Dongcheng Liu, Xueqiang Zhao, Aimin Zhang, Yiping Tong

**Affiliations:** ^1^State Key Laboratory of Wheat and Maize Crop Science, College of Agronomy, Collaborative Innovation Center of Henan Grain Crops, Henan Agricultural University, Zhengzhou, China; ^2^China Agricultural University, Beijing, China; ^3^State Key Laboratory for Plant Cell and Chromosome Engineering, Institute of Genetics and Developmental Sciences, Chinese Academy of Sciences, Beijing, China

**Keywords:** *Triticum aestivum* L., quantitative trait locus (QTL), root traits, nitrogen deficiency, phosphorus deficiency

## Abstract

Root is important in acquiring nutrients from soils. Developing marker-assisted selection for wheat root traits can help wheat breeders to select roots desirable for efficient acquisition of nutrients. A recombinant inbred line (RIL) population derived from wheat varieties Xiaoyan 54 and Jing 411 was used to detect QTLs for maximum root length and root dry weight (RDW) under control, low nitrogen and low phosphorus conditions in hydrophobic culture (HC). We totally detected 17 QTLs for the investigated root traits located at 13 loci on 11 chromosomes. These loci differentially expressed under different nutrient supplying levels. The RILs simultaneously harboring positive alleles or negative alleles of the most significant three QTLs for RDW, *qRDW.CK-2A, qRDW.CK-2D*, and *qRDW.CK-3B*, were selected for soil column culture (SC) trial to verify the effects of these QTLs under soil conditions. The RILs pyramiding the positive alleles not only had significantly higher shoot dry weight, RDW, nitrogen and phosphorus uptake in all the three treatments of the HC trial, but also had significantly higher RDW distribution in both the top- and sub-soils in the SC trial than those pyramiding the negative alleles. These results suggested that QTL analysis based on hydroponic culture can provide useful information for molecular design of wheat with large and deep root system.

## Introduction

High crop yield largely depends on input of water and fertilizers. During 1960–2000 of the last century, the world food production nearly doubled, but the annual consumption of nitrogen (N) and phosphorus (P) fertilizers increased 7.8-folds (from 10 to 88 Tg) and 3.4-folds (from 9 to 40 Tg), respectively; and the irrigated land increased from 10 to 18% of arable land (Vance, 2001). The high input has led to degradation of soil, air and water quality, and the exhaustion of natural resources such as water and nutrients (Holford, 1997; Vance et al., 2003; Guo et al., 2010). Moreover, producing more food for the growing population will put further enormous pressure on natural resources and environments. Therefore, it is needed to improve resource use efficiency of crops in the future to minimize the negative impact of increasing crop yield on natural resources and environments.

In the past, the development of high-yield varieties of cereal crops were mostly achieved by selection of the above-ground organs (e.g., the first green revolution), and the below-ground roots were largely neglected (Waines and Ehdaie, 2007). Considering the importance of roots in determining acquisition efficiency of soil resources like nutrients and water, manipulating root traits such as RSA and nutrient uptake has been suggested to enable a very much needed new green revolution and further increase in yields (de Dorlodot et al., 2007; Lynch, 2007; Den Herder et al., 2010). However, roots, the hidden half of a plant, are difficult to be selected directly by breeders, thus developing marker-assisted selection (MAS) will help breeders to select root traits desirable for efficient acquisition of water and nutrients from soils. QTL analysis has observed overlap between QTLs for root traits and those for nutrient uptake and productivity in many crop species such as wheat (Sharma et al., 2011; Xie et al., 2017; Yuan et al., 2017), rice (Steele et al., 2007; Uga et al., 2013), maize (Tuberosa et al., 2002; Hochholdinger and Tuberosa, 2009), and soybean (Liang et al., 2010), suggesting the valuable potential of marker-assisted breeding for root traits in increasing resource use efficiency and yields.

Wheat is one of the most important food crops in the world; thus identifying QTLs toward marker-assisted breeding for root traits desirable for efficient acquisition of soil nutrients may offer a sustainable solution to nutrient management in wheat production. It is well known that root development is susceptible to nutrient availability, therefore understanding genetic basis of roots responding to nutrient availability is of significance in breeding wheat with root system desirable for nutrient uptake in a wide nutrient regime. A number of literatures have reported QTLs for root biomass and morphological parameters in wheat (Laperche et al., 2006; Sanguineti et al., 2007; Canè et al., 2014; Atkinson et al., 2015; Maccaferri et al., 2016; Iannucci et al., 2017). However, the most challenge in identifying QTLs for root traits is the difficulty in observing roots grown in the soils, especially when analyzing a large number of genotypes in QTL mapping. Up to now, root QTLs were chiefly detected by using hydroponic culture or sand culture. Although QTLs for root characteristics detected in hydroponics have been reported to coincide with nutrient uptake and yield components by wheat (An et al., 2006; Canè et al., 2014; Horn et al., 2016) in field trials, whether QTLs expressed in hydroponics can be used in selecting root traits of soil-grown wheat plants is still uncertain.

In order to clarify this problem, we designed the following experiments. Firstly, we mapped QTLs for root traits and analyzed their relations with nitrogen (N) and phosphorus (P) uptake under different nitrogen and phosphorus supply levels in a hydroponic culture using a recombinant inbred line (RIL) population. Furthermore, the RILs simultaneously harboring positive alleles (8 lines) or negative alleles (11 lines) of the top three QTLs for RDW in phenotypic contribution rate were selected for soil culture (SC) trial to verify the effects of these QTLs under soil conditions. The results showed that QTL analysis based on hydroponic culture can provide practical information for molecular design of wheat with large and deep root system and efficient nutrient uptake.

## Materials and methods

### Plant materials

One recombinant inbred line (RIL) population was used in this study. The RIL population contained 142 RILs derived from two Chinese winter wheat varieties Xiaoyan 54 and Jing 411. Xiaoyan 54 has been shown to have higher efficiency in acquisition of nitrogen from soil than Jing 411, regardless of the soil depth, consistent with the finding that Xiaoyan 54 had a bigger and deeper root system than Jing 411 (Zhang et al., 2005).

### Root morphology evaluation

A hydroponic culture (HC) experiment was carried out to phenotype the RIL population at seedling stage. In the hydroponic culture, seeds were germinated in saturated CaSO_4_ solution for 7 days at 20°C, and then the germinated seeds with residual endosperm removed were transferred to three tanks (2.5 m long × 1.5 m wide × 0.5 m high) each containing 1,800 L of nutrient solution as described previously (Li et al., 2011; Figure S1). The tanks contained sufficient full strength (CK), low nitrogen (N), and low phosphorus (P) nutrient solution, respectively. The CK nutrient solution contained (mM) Ca(NO_3_)_2_ 1, KH_2_PO_4_ 0.2, MgSO_4_ 1, KCl 1.5, CaCl_2_ 1.5, H_3_BO_3_ 1 × 10^−3^, (NH_4_)_6_Mo_7_O_24_ 5 × 10^−5^, CuSO_4_ 5 × 10^−4^, ZnSO4 1 × 10^−3^, MnSO4 1 × 10^−3^, Fe(III)–EDTA 1 × 10^−1^. The low N (LN) nutrient solution contained the same nutrients concentration with full strength nutrient solution except Ca(NO_3_)_2_ (0.1 mM) and CaCl_2_ (2.4 mM). The low P (LP) nutrient solution contained the same nutrients concentration with full strength nutrient solution except KH_2_PO_4_ (0.01 mM) and KCl (1.69 mM). The pH of nutrition solution was adjusted to 6.0 with dilute HCl and NaOH before transferring. The RILs were randomly placed and grown in a greenhouse with three replications each. About 450 plants were grown in each tank. The solution was changed every 7 days. The lowest temperature of the greenhouse ranged from 5 to 10°C, and the highest temperature varied from 18 to 30°C of each day during the period of HC trial. The root morphologies and other related traits of the “Xiaoyan 54 × Jing 411” RIL population were investigated after these lines were grown for 35 days in the hydroponic trial. The developmental stages of the parents of the RIL population, Xiaoyan54 and J411, were Zadoks growth scale 24 and 23, respectively under CK condition; while the developmental stage of Xiaoyan54 and J411 were ranged from Zadoks growth scale 21 to 22 under LN and LP conditions (Zadoks et al., 1974).

To verify whether QTLs expressed in hydroponics can be used in selecting root traits of soil-grown wheat plants, The RILs simultaneously harboring positive (8 lines) or negative alleles (11 lines) of the top three QTLs for RDW in phenotypic contribution rate were selected as materials to conduct a soil column culture (SC) experiment to phenotype the root traits and nutrient (N, P) use efficiency under soil culture condition. The SC trial was carried out in the experimental station of Institute of Genetics and Developmental Biology, Chinese Academy of Sciences in Beijing. Before sowing, 87.8 mg KH_2_PO_4_, 107.14 mg urea and 4.4 mg ZnSO_4_.7H_2_O were well mixed with every 1 kilogram soil used in this trial. Seeds were germinated for 24 h at 20°C, and then five germinated seeds were sowed in each PVC tube with 90 cm-high and 11 cm-diameter. Wheat plants were thinned to three plants per tube at the 2nd leaf developmental stage (Zadoks growth scale 12) (Zadoks et al., 1974). The sowing date was September 27, 2014, which was the suitable time for winter wheat sowing in field in Beijing. The soil columns were randomly placed and with three replications each. The lowest temperature ranged from 5 to 12°C, and the highest temperature varied from 15 to 27°C of each day during the period of SC trial. Plants were harvested 35 days after germination. The roots were washed free of sand to evaluate root traits.

In the hydroponic culture trial, the maximal root length (MRL), root dry weight (RDW), tiller (TN), shoot dry weight (SDW), were investigated under sufficient nutrient supply, low N and low P conditions; while in the soil column trial, the root dry weight distribution in the soil profiles 0–30 cm (RDW1), 30–60 cm (RDW2), >60 cm (RDW3), TN, SDW were investigated under sufficient nutrient condition. Total N and P concentration in root and shoot tissues were measured according to previous reported literatures (An et al., 2006; Su et al., 2006) in both trials. The N (NUP) and P (PUP) uptake were calculated as the sum of N or P accumulated in root (RDW × root N (P) concentration) and shoot (SDW × Shoot N (P) concentration).

### Statistical analysis

The means, standard deviations, standard errors and ranges of each measured morphological traits were calculated by using SPSS11.5 software. The statistical significance of differences across the RILs and the two parents were analyzed by using Fischer LSD (least-significant difference) test for all of the traits, with *P*-value < 0.05 considered significant. The correlations between different traits were also analyzed by using SPSS11.5 software.

### QTL detection

The genetic map of the “Xiaoyan 54 × Jing 411” RIL population was described by Ren et al. (2012). The composite interval mapping was applied for QTL mapping (Zeng, 1994). In the forward regression analysis, the walk speed and window size were set as 2 and 10 cM, respectively, with five control markers. The phenotypic variation explained by a single QTL was determined by the square of the partial correlation coefficient (*R*^2^). Analyses of QTL location, additive effect and 95% confidence intervals of QTLs were performed using WinQTLCart 2.5 software (Model 6, Basten et al., 2001). The threshold of LOD value for QTL detection was set as 2.5.

## Results

### Evaluation of phenotypes

We evaluated RDW and MRL under CK, low N and low P conditions. As shown in Table 1, the female parent Xiaoyan 54 had similar RDW and MRL with the male parent Jing 411 under CK condition, but it had higher RDW and longer MRL than Jing 411 under both low N and low P conditions. Low N and low P treatments increased RDW and MRL of the female parent Xiaoyan 54, but did not significantly influenced on that of Jing 411 (except that low N reduced MRL in Jing 411) (Table 1). The differential response of RDW and MRL to low N and low P stresses also existed among the RILs (Table S1). The RIL lines showed large variations in all the investigated traits (Table 1). There existed RILs with values that were higher or lower than both parents in all the evaluated traits, indicating potential transgressive variations and the presence of positive and negative alleles in both parents.

**Table 1:** Mean values and ranges for root traits (RDW and MRL) in the RIL population and their parents under CK, low N and low P conditions.

**Trait**	**Treatment**	**Parent (Mean** ± **SE)**		**RIL**		
		**Xiaoyan54**	**Jing 411**	**Mean ± SD**	**Min**.	**Max**.
RDW	CK	0.071 ± 0.020	0.078 ± 0.010	0.067 ± 0.019	0.030	0.128
	Low N	0.107 ± 0.011 (A)	0.084 ± 0.008 (B)	0.109 ± 0.027	0.056	0.208
	Low P	0.121 ± 0.014 (A)	0.083 ± 0.001 (B)	0.092 ± 0.021	0.044	0.140
MRL	CK	41.3 ± 4.4	39.3 ± 3.7	36.7 ± 8.7	14.7	55.0
	Low N	54.3 ± 2.4 (A)	29.7 ± 7.2 (B)	42.1 ± 10.0	16.0	65.3
	Low P	51.5 ± 0.4 (A)	38.7 ± 5.8 (B)	38.6 ± 10.9	18.3	64.0

*RDW, root dry weight (g plant^−1^); MRL, maximum root length (cm)*.

*Statistical difference between the two parents is indicated by different letters after the means. Capital letters designate significance at P < 0.01*.

We also investigate the above-ground traits (TN and SDW) and nutrient uptake (NUP and PUP). These four traits exhibited large variations among the RIL lines (Table S2). There were significantly and positively correlation coefficients between TN, SDW, RDW NUP and PUP in all the three treatments (Table S3), indicating that wheat lines with vigorous roots and shoots could facilitate N and P uptake. Generally, MRL showed relative poorer correlations with other investigated traits (Table S3).

### Identification of QTLs for root traits

**Figure 1:**
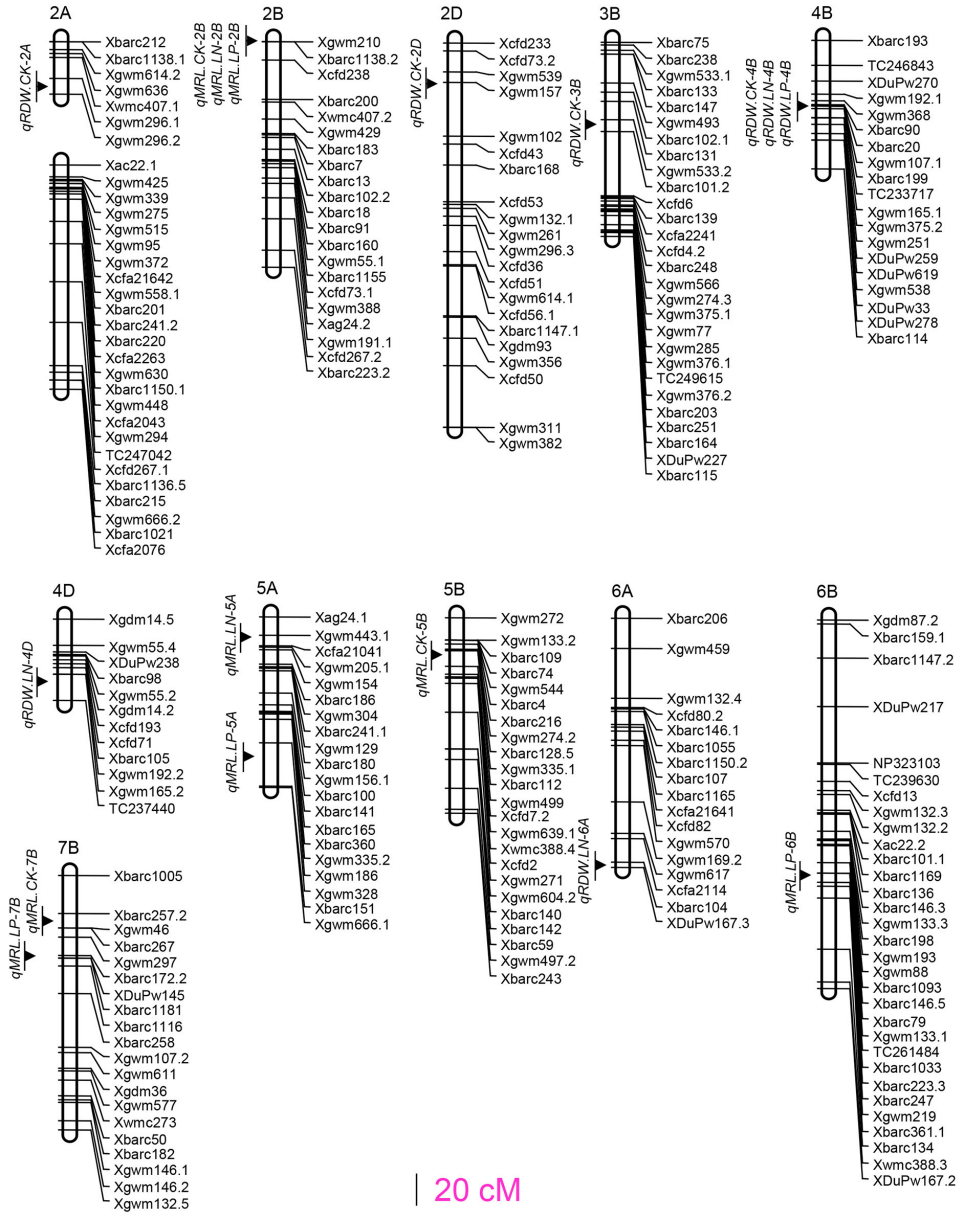
QTLs for the investigated root traits under CK, low N and low P conditions.

The trial detected eight QTLs for total root dry weight (RDW) and nine QTLs for maximum root length (MRL), respectively. These 17 QTLs located at 13 loci on 11 chromosomes (Figure 1). The locus on chromosomes 2D (*Xgwm539*-*Xgwm157*) controlled RDW under all the three treatments, the locus on chromosomes 2B (*Xgwm210*-*Xbarc1138.2*) associated with MRL under all the three treatments (Figure 1; Table 2), and one locus (*Xbarc257.2* and *Xbarc1116* on chromosome 7B) controlled MRL under CK and low P conditions. Four loci (*qRDW.CK-2A, qRDW.CK-2D, qRDW.CK-3B*, and *qMRL.CK-5B*) were detected to regulate root traits under control condition specifically, three loci (*qRDW.LN-4D, qMRL.LN-5A*, and *qRDW.LN-6A*) were specifically for root traits under low N condition, two loci (*qMRL.LP-5A* and *qMRL.LP-6B*) were specifically expressed under low P condition (Figure 1; Table 2).

**Table 2:** Detected QTLs for root traits under CK, low N and low P conditions.

**Trait**	**Treatment**	**QTL**	**Chr^a^**	**Marker interval^b^**	**LOD^c^**	***R*^2^ × 100**	**Additive^d^**
RDW (g/plant)	CK	*qRDW.CK-2A*	2A	*Xgwm296.1-*Xgwm296.2**	2.7	8.9	−0.006
	CK	*qRDW.CK-2D*	2D	*Xgwm157-Xgwm102*	3.8	15.3	0.008
	CK	*qRDW.CK-3B*	3B	*Xgwm533.2-Xbarc101.2*	3.3	10.9	−0.006
	CK	*qRDW.CK-4B*	4B	*Xbarc90-Xbarc20*	3.4	7.9	−0.006
	Low N	*qRDW.LN-4B*	4B	*Xbarc90-Xbarc20*	4.7	10.4	−0.009
	Low N	*qRDW.LN-4D*	4D	*Xgwm165.2- TC237440*	2.9	8.8	0.008
	Low N	*qRDW.LN-6A*	6A	*Xbarc104-Xdwpw167.3*	3.0	8.6	−0.008
	Low P	*qRDW.LP-4B*	4B	*TC233717-Xgwm165.1*	4.1	9.6	−0.007
MRL (cm)	CK	*qMRL.CK-2B*	2B	*Xgwm210-Xbarc1138.2*	12.8	32.7	5.1
	CK	*qMRL.CK-5B*	5B	*Xbarc112-Xgwm499*	3.5	6.5	−2.3
	CK	*qMRL.CK-7B*	7B	*Xbarc257.2-Xgwm46*	3.8	10.4	2.9
	Low N	*qMRL.LN-2B*	2B	*Xgwm210-Xbarc1138.2*	6.5	21.6	4.7
	Low N	*qMRL.LN-5A*	5A	*Xgwm443.1-Xcfa21041*	2.7	6.8	−2.7
	Low P	*qMRL.LP-2B*	2B	*Xgwm210-Xbarc1138.2*	5.4	16.4	4.5
	Low P	*qMRL.LP-5A*	5A	*Xgwm328-Xbarc151*	3.2	15.2	−4.3
	Low P	*qMRL.LP-6B*	6B	*TC261484-Xbarc1033*	2.5	5.4	2.6
	Low P	*qMRL.LP-7B*	7B	*Xbarc1181-Xbarc1116*	3.5	7.7	3.2

a *Chr means chromosome name*.

b *Markers underlined were the nearest marker to the QTL*.

c *LOD means Logarithm of odds*.

d *Additive effects, a positive sign means that positive allele comes from the parent Xiaoyan 54, while a negative sign means positive allele comes from the parent Jing 411*.

### Effects of pyramiding QTLs on RDW and nutrient uptake

We then analyzed the effects of pyramiding QTLs on RDW and nutrient uptake. We analyzed the effects of pyramiding the QTLs for RDW detected under CK and LN conditions, respectively. Firstly, we pyramided the three most significant QTLs of the five QTLs for RDW detected under CK condition (*qRDW.HC.CK-2A, qRDW.CK-2D*, and *qRDW.CK-3B*). The positive pyramiding (the RILs simultaneously harboring the positive alleles at these three QTLs) averagely had 81.0, 64.4, and 55.5% higher RDW than the negative pyramiding (the RILs simultaneously harboring the negative alleles) under CK, LN and LP conditions, respectively (Table 3). Moreover, the positive pyramiding QTLs for RDW significantly increased SDW, NUP and PUP in all the treatments (Table 3). Similarly, the positive pyramiding of the three QTLs for RDW detected under LN condition, *qRDW.LN-4B, qRDW.LN-4D*, and *qRDW.LN-6A*, averagely had 42.3, 46.7, and 29.6% higher RDW than the negative pyramiding under CK, LN and LP conditions, respectively (Table S4), and increased SDW, NUP, and PUP significantly in all the treatments (Table S4).

**Table 3:** Pyramiding QTLs for root biomass enhanced N and P uptake and TGW.

**Trait**	**Treatment**	***Xgwm296.2-2A** + **Xgwm157-2D** + **Xgwm533.2-3B***		**Increase (%)**
		**Positive (*n* = 11)**	**Negative (*n* = 8)**	
**HYDROPONIC CULTURE**				
TN (tiller/plant)	CK	2.90 ± 0.67	2.76 ± 0.68	5.0
	LN	2.19 ± 0.59	2.09 ± 0.64	4.6
	LP	1.83 ± 0.55	1.65 ± 0.51	11.0
SDW (g/plant)	CK	0.454 ± 0.086 (A)	0.277 ± 0.054 (B)	63.7
	LN	0.353 ± 0.063 (A)	0.232 ± 0.044 (B)	52.6
	LP	0.312 ± 0.055 (A)	0.210 ± 0.033 (B)	49.0
RDW (g/plant)	CK	0.085 ± 0.020 (A)	0.047 ± 0.008 (B)	81.0
	LN	0.138 ± 0.033 (A)	0.084 ± 0.025 (B)	64.4
	LP	0.107 ± 0.025 (A)	0.069 ± 0.011 (B)	55.5
MRL (cm)	CK	34.9 ± 8.6	28.7 ± 9.4	21.5
	LN	41.7 ± 8.8	35.1 ± 10.7	18.7
	LP	31.9 ± 8.9	30.6 ± 7.1	4.4
NUP (mg/plant)	CK	23.4 ± 4.4 (A)	14.4 ± 2.9 (B)	63.0
	LN	9.8 ± 1.6 (A)	6.9 ± 1.2 (B)	41.9
	LP	12.2 ± 2.0 (A)	9.0 ± 1.4 (B)	35.3
PUP (mg/plant)	CK	4.57 ± 0.85 (A)	3.07 ± 0.62 (B)	48.7
	LN	3.60 ± 0.71 (A)	2.51 ± 0.56 (B)	43.4
	LP	0.64 ± 0.11 (A)	0.50 ± 0.09 (B)	28.3
**SOIL COLUMN CULTURE**				
TN (tiller/plant)		8.8 ± 0.8	8.7 ± 1.3	0.3
SDW (g/plant)		1.004 ± 0.111 (a)	0.830 ± 0.149 (b)	21.0
RDW (g/plant)	Total	0.294 ± 0.053 (A)	0.227 ± 0.043 (B)	29.6
	0–30 cm	0.163 ± 0.026 (a)	0.130 ± 0.025 (b)	24.9
	30–60 cm	0.064 ± 0.014 (A)	0.044 ± 0.012 (B)	46.4
	>60 cm	0.068 ± 0.015 (a)	0.053 ± 0.012 (b)	28.3
MRL (cm)		122.3 ± 6.5	120.3 ± 8.4	1.6
NUP (mg/plant)		52.2 ± 6.3 (A)	42.5 ± 7.3 (B)	22.7
PUP (mg/plant)		5.36 ± 0.58 (a)	4.44 ± 0.91 (b)	20.8

*Statistical difference between the positively and the negatively pyramiding groups is indicated by different letters after the means. Capital and small letters designate significance at P < 0.01 and P < 0.05, respectively*.

To verify the effects of these QTLs under soil conditions, lines simultaneously harboring the positive alleles or negative alleles of the top three QTLs for RDW detected under CK condition were selected for soil culture trial. Also, the positive pyramiding averagely had significantly higher total RDW and root dry weight at different soil layers, and higher nutrient uptake than the negative pyramiding in the SC trial (Table 3).

## Discussion

The current study was carried out to detect QTLs for root growth under different N and P supply levels. The “Xiaoyan 54 × Jing 411” RIL population was used in the present study as it has been shown to segregate in root morphology (Ren et al., 2012). Although Xiaoyan 54 and Jing 411 showed no differences in RDW and MRL under CK condition in the HC trial, Xiaoyan 54 had significant higher RDW and longer MRL than Jing 411 under both low N and low P conditions (Table 1), indicating the root growth of Xiaoyan 54 more strongly responded to N- and P-deficiency than that of Jing 411.

Our current study observed large differences among the RILs of the “Xiaoyan 54 × Jing 411” RIL population of all the investigated root traits (Table 1). Both RDW and MRL showed positively and significantly correlations with NUP and PUP in all the treatments (Table S3). These results demonstrated the importance of enhancing root development in improving N and P use efficiencies whatever under low N, low P or CK condition, confirming the previously reported results that early vigorous growth improves nitrogen and phosphate uptake in wheat (Liao et al., 2004; Ehdaie et al., 2010; Ryan et al., 2015; Wang et al., 2016).

We totally detected 17 QTLs for the investigated root traits located at 13 loci on 11 chromosomes (Figure 1). Pyramiding QTLs for RDW detected in the HC trial not only largely increased RDW, NUP and PUP in the HC trial (Table 3; Table S4), but also significantly increased total RDW and root distribution in both the top- and sup-soils, and consequently enhanced nutrient uptake and shoots growth in the SC trial (Table 3). This result suggested that QTL analysis based on hydroponic culture can provide practical information for molecular design of wheat with large and deep root system. In fact, these three QTLs (*qRDW.CK-2A, qRDW.CK-2D*, and *qRDW.CK-3B*) associated with QTLs for root growth or nutrient uptake detected in the current study and reported previously. The QTL *qRDW.CK-2A* linked with *qSDW.LP-2A, qNUP.LP-2A* and *qPUP.LP-2A* (Figure S2) and a QTL for P uptake in a P-deficient soil at seedling stage (Su et al., 2006). The QTL *qRDW.CK-2D* coincided with seven QTLs for RDW, SDW, NUP, and PUP (Figure S2; Table S5), and RDW at seedling stage, NUP and PUP investigated at seedling stage and maturity in different N and P availabilities (An et al., 2006; Su et al., 2009). The QTL *qRDW.CK-3B* coincided with QTLs for SDW under CK and low N conditions, NUP and PUP under CK condition (Figure S2; Table S5), and associated with QTLs for SDW and PUP investigated at seedling stage and maturity in P-deficient or sufficient soil (Su et al., 2009). The QTLs *qRDW.CK-2A* and *qRDW.CK-2D* also linked with N-use efficient meta-QTL-1 and –2, respectively (Quraishi et al., 2011). These results further supported the importance of these three loci in breeding wheat with improved root growth and nutrient uptake.

Other loci for root growth mapped in the present study also show potential in breeding root growth and nutrient uptake. The QTLs *qRDW.CK-4B, qRDW.LN-4B*, and *qRDW.LP-4B* were located on the adjacent chromosome region and coincided with a number of QTLs for SDW, PUP and NUP under CK, low N and low P conditions (Figure S2; Table S5). In previous studies using “Xiaoyan 54 × Jing 411” RIL population, this chromosomal region was identified to affect plant height, harvest index under CK, low N and low P conditions, and shoot N content under low N condition at adult stages in field trials (Xu et al., 2014), and shoot height and biomass production at the seedling stage in both salt stress and control treatments (Xu et al., 2012). In this study, they were also detected to have pleiotropic effects for RDW, SDW, NUP, and PUP traits. Actually, this chromosomal region has been reported controlling RDW and SDW under CK condition, and biomass under Low P condition in previous published literatures (Ryan et al., 2015; Maccaferri et al., 2016; Iannucci et al., 2017), and is coincident with that of the *Rht-B1* gene, which is the main locus involved in the control of plant height (Börner et al., 1996). It was not clear whether *Rht-B1* gene to be separated from the QTL for RDW or not. Importantly, this chromosomal region was identified controlling both shoot and root traits at seedling and adult stages, and across various environments, which further confirmed that it has non-environment-specific and non-stage-specific genes (Xu et al., 2014).

On chromosome 2B, we mapped a major locus between markers *Xgwm210* and *Xbarc1138.2* explained 32.7, 21.6, and 16.4% of MRL phenotypic variations under CK, low N and low P conditions, respectively (Figure 1; Table 2). In fact, this locus has been reported controlling multi-root morphologic parameters (Ren et al., 2012) and yield components (Hai et al., 2008). The constitutively expression under different N and P supply levels in this research and its association with QTL for grain yield components suggested the importance of this locus in improving wheat nutrients use efficiency and yield.

Among the 13 loci for root traits detected in the current study, three loci (*qRDW.LN-4D, qRDW.LN-6A*, and *qMRL.LN-5A*) specifically expressed under low N condition and two loci (*qMRL.LP-5A* and *qMRL.LP-6B*) specifically expressed under low P condition (Figure 1). *qRDW.LN-6A* specifically expressed under low N condition and coincided with several previously reported QTLs for KNS (Kernel number per spike), HI (Harvest index) and TKW (Thousand kernel weight) under low N condition. *qMRL.LP-5A* specifically expressed under low P condition and coincided with previously reported QTLs for DM (Dry matter) under CK and low P condition (Xu et al., 2014). *qMRL.LP-6B* was tightly linked with previously reported QTLs for root length (Maccaferri et al., 2016; Iannucci et al., 2017), indicating the sustainable expression in different genetic background. These loci might be useful in improving the adaptability of root system to low nutrient availability.

In summary, this work detected 17 QTLs for root traits located at 13 loci on 11 chromosomes under different N and P Supply levels. Encouragingly, we found that QTL analysis of root traits based on hydroponic culture can provide valuable clues for the development of wheat varieties with large and deep root system through QTL pyramiding. Considering the complexity of the genetic basis of root traits, more studies are needed to further validate these regions as targets for genetic improvement of root traits in field conditions.

## Author contributions

YR and YQ performed the experiments; DL and AZ were responsible for DNA markers analysis and linkage analysis; YR and YX contributed to genetic mapping and QTL analysis; XZ, YT, and CZ conceived and designed the experiments; YR and YT wrote the paper.

### Conflict of interest statement

The authors declare that the research was conducted in the absence of any commercial or financial relationships that could be construed as a potential conflict of interest.

## Supplementary material

Figure S1 The hydroponic equipment utilized in this work. The length, width and height of the hydroponic equipment are 2.5, 1.5, and 0.5 m, respectively.

Figure S2 QTLs for the investigated root traits, tiller number, shoot dry weight, nitrogen, and phosphorus uptake under CK, low N and low P conditions.

Table S1 The values of root dry weight and maximum root length of the recombinant inbred lines under CK, low N, and low P conditions.

Table S2 Mean values and ranges for tiller number, shoot dry weight, nitrogen and phosphorus uptake in the RIL population and their parents under CK, low N, and low P conditions.

Table S3 Correlation coefficients between the traits under CK, low N, and low P conditions.

Table S4 Pyramiding QTLs for root biomass detected under low nitrogen condition enhanced N and P uptake.

Table S5 Detected QTLs for tiller number, shoot dry weight, nitrogen and phosphorus uptake.
