# Supplementary material for: Characterization of QTLs for Root Traits of Wheat Grown under Different Nitrogen and Phosphorus Supply Levels
Source: Front Plant Sci. 2017 Dec 11;8:2096. doi: 10.3389/fpls.2017.02096 (PMC5732362; doi:10.3389/fpls.2017.02096)
Supplement: Figure S1 — The hydroponic equipment utilized in this work. The length, width and height of the hydroponic equipment are 2.5, 1.5, and 0.5 m, respectively. [file Presentation1.PPT]

## Slide 1
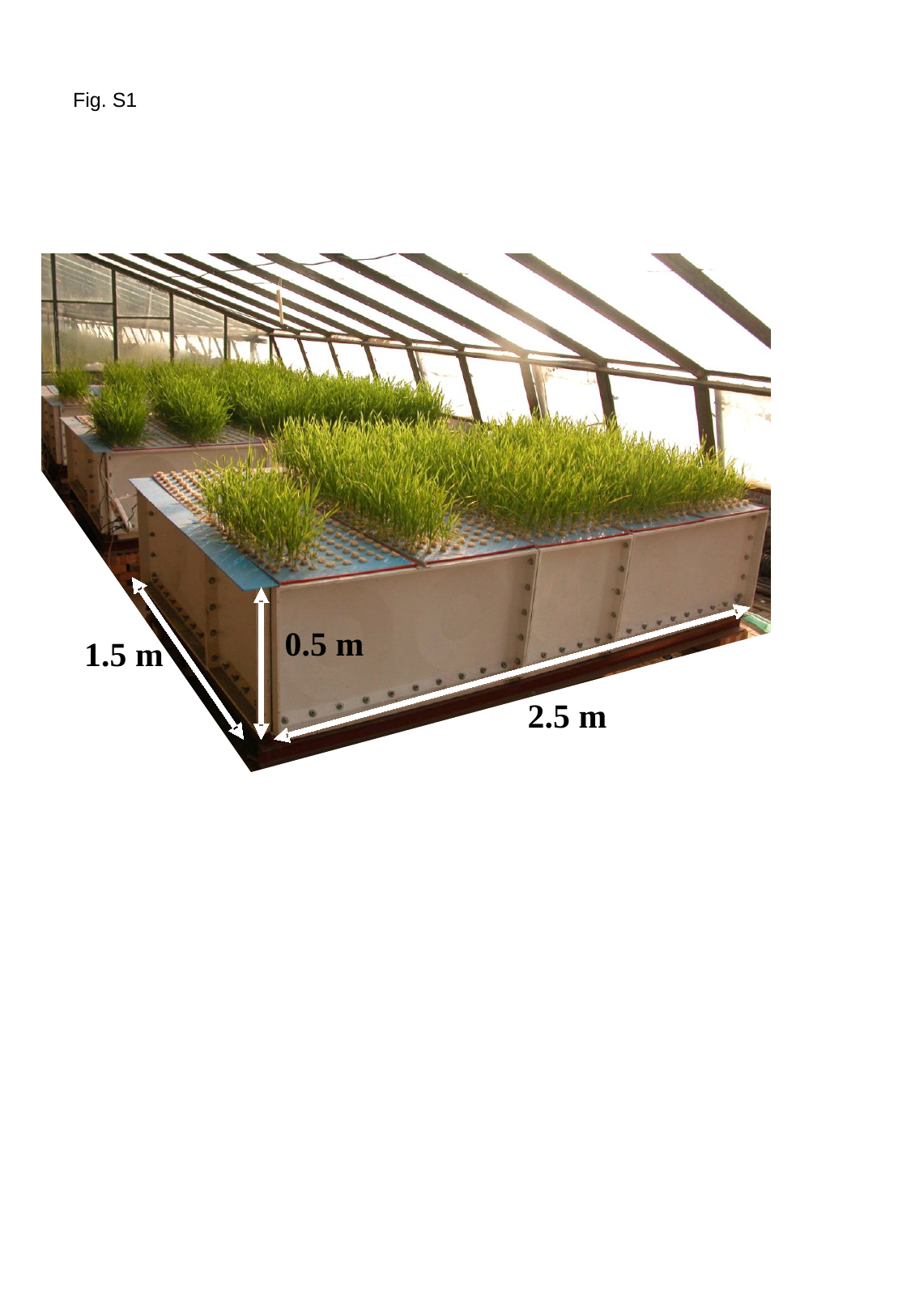

Fig. S1
0.5 m
1.5 m
2.5 m

## Slide 2
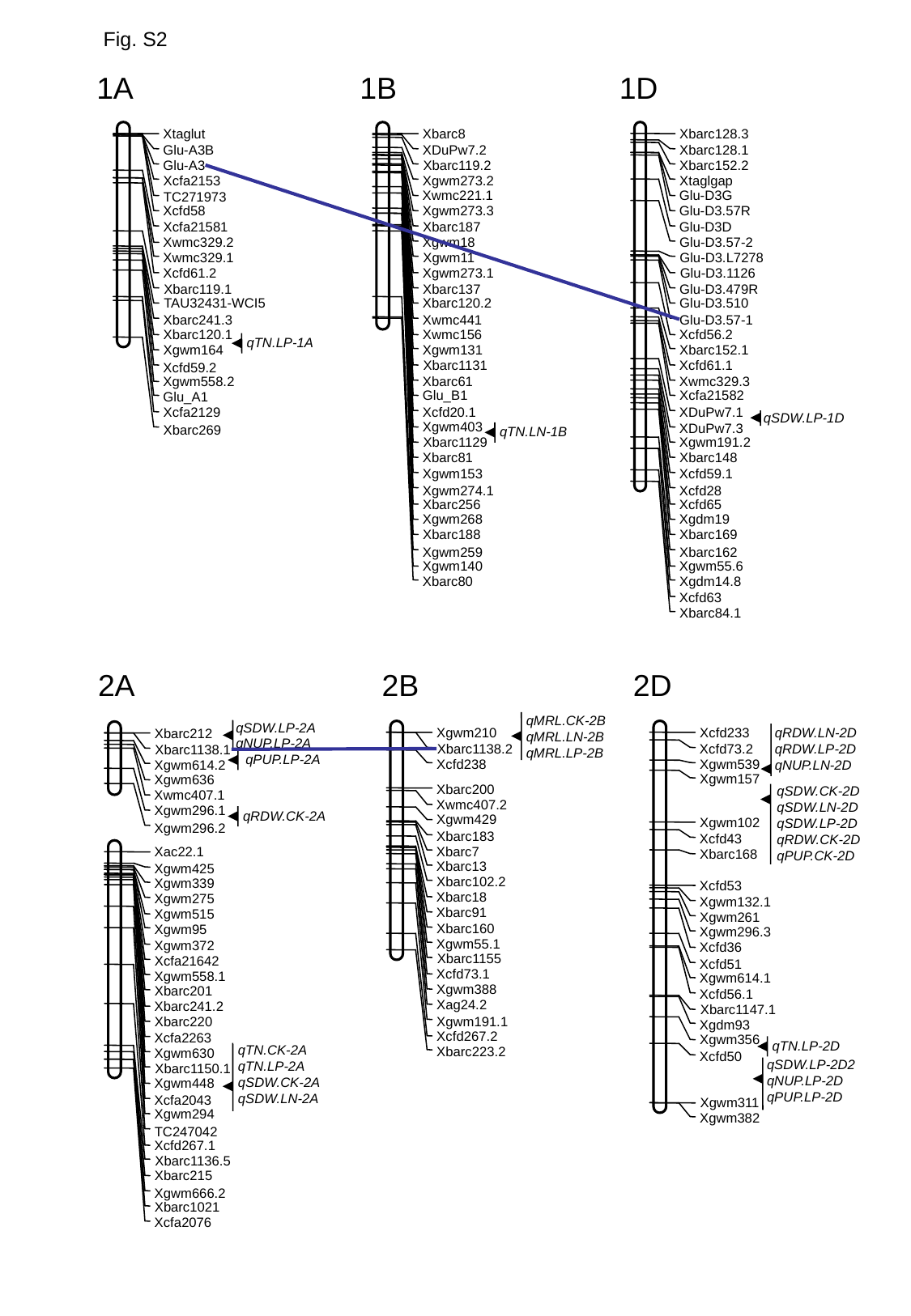

Fig. S2
1A
Xtaglut
Glu-A3B
Glu-A3
Xcfa2153
TC271973
Xcfd58
Xcfa21581
Xwmc329.2
Xwmc329.1
Xcfd61.2
Xbarc119.1
TAU32431-WCI5
Xbarc241.3
Xbarc120.1
Xgwm164
Xcfd59.2
Xgwm558.2
Glu_A1
Xcfa2129
Xbarc269
qTN.LP-1A
1B
Xbarc8
XDuPw7.2
Xbarc119.2
Xgwm273.2
Xwmc221.1
Xgwm273.3
Xbarc187
Xgwm18
Xgwm11
Xgwm273.1
Xbarc137
Xbarc120.2
Xwmc441
Xwmc156
Xgwm131
Xbarc1131
Xbarc61
Glu_B1
Xcfd20.1
Xgwm403
Xbarc1129
Xbarc81
Xgwm153
Xgwm274.1
Xbarc256
Xgwm268
Xbarc188
Xgwm259
Xgwm140
Xbarc80
qTN.LN-1B
1D
Xbarc128.3
Xbarc128.1
Xbarc152.2
Xtaglgap
Glu-D3G
Glu-D3.57R
Glu-D3D
Glu-D3.57-2
Glu-D3.L7278
Glu-D3.1126
Glu-D3.479R
Glu-D3.510
Glu-D3.57-1
Xcfd56.2
Xbarc152.1
Xcfd61.1
Xwmc329.3
Xcfa21582
XDuPw7.1
XDuPw7.3
Xgwm191.2
Xbarc148
Xcfd59.1
Xcfd28
Xcfd65
Xgdm19
Xbarc169
Xbarc162
Xgwm55.6
Xgdm14.8
Xcfd63
Xbarc84.1
qSDW.LP-1D
2A
qSDW.LP-2A
qNUP.LP-2A
Xbarc212
Xbarc1138.1
Xgwm614.2
Xgwm636
Xwmc407.1
Xgwm296.1
Xgwm296.2
Xac22.1
Xgwm425
Xgwm339
Xgwm275
Xgwm515
Xgwm95
Xgwm372
Xcfa21642
Xgwm558.1
Xbarc201
Xbarc241.2
Xbarc220
Xcfa2263
Xgwm630
Xbarc1150.1
Xgwm448
Xcfa2043
Xgwm294
TC247042
Xcfd267.1
Xbarc1136.5
Xbarc215
Xgwm666.2
Xbarc1021
Xcfa2076
qPUP.LP-2A
qRDW.CK-2A
qTN.CK-2A
qTN.LP-2A
qSDW.CK-2A
qSDW.LN-2A
2B
qMRL.CK-2B
qMRL.LN-2B
qMRL.LP-2B
Xgwm210
Xbarc1138.2
Xcfd238
Xbarc200
Xwmc407.2
Xgwm429
Xbarc183
Xbarc7
Xbarc13
Xbarc102.2
Xbarc18
Xbarc91
Xbarc160
Xgwm55.1
Xbarc1155
Xcfd73.1
Xgwm388
Xag24.2
Xgwm191.1
Xcfd267.2
Xbarc223.2
2D
qRDW.LN-2D
qRDW.LP-2D
qNUP.LN-2D
Xcfd233
Xcfd73.2
Xgwm539
Xgwm157
Xgwm102
Xcfd43
Xbarc168
Xcfd53
Xgwm132.1
Xgwm261
Xgwm296.3
Xcfd36
Xcfd51
Xgwm614.1
Xcfd56.1
Xbarc1147.1
Xgdm93
Xgwm356
Xcfd50
Xgwm311
Xgwm382
qSDW.CK-2D
qSDW.LN-2D
qSDW.LP-2D
qRDW.CK-2D
qPUP.CK-2D
qTN.LP-2D
qSDW.LP-2D2
qNUP.LP-2D
qPUP.LP-2D

## Slide 3
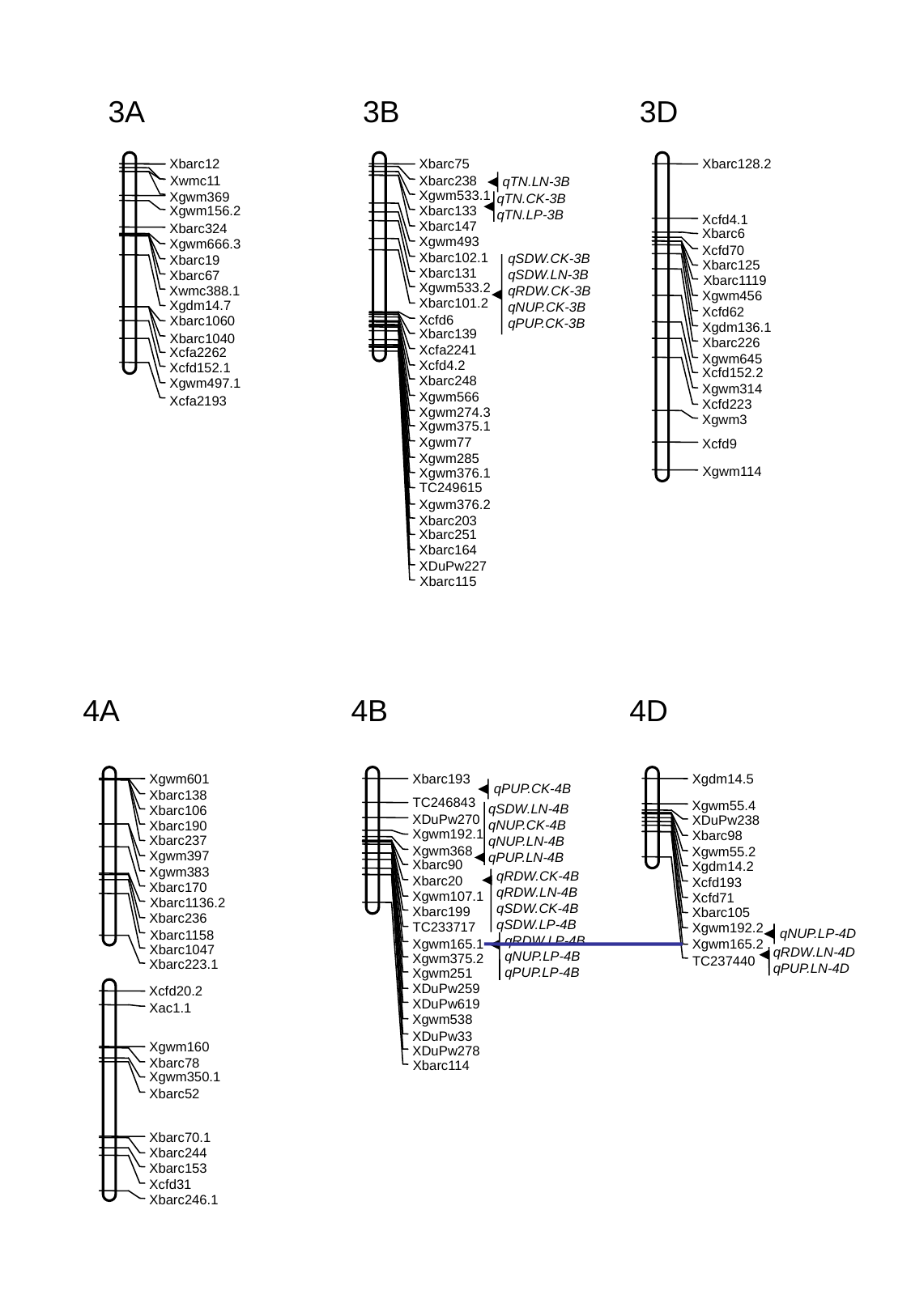

3A
Xbarc12
Xwmc11
Xgwm369
Xgwm156.2
Xbarc324
Xgwm666.3
Xbarc19
Xbarc67
Xwmc388.1
Xgdm14.7
Xbarc1060
Xbarc1040
Xcfa2262
Xcfd152.1
Xgwm497.1
Xcfa2193
3B
Xbarc75
Xbarc238
Xgwm533.1
Xbarc133
Xbarc147
Xgwm493
Xbarc102.1
Xbarc131
Xgwm533.2
Xbarc101.2
Xcfd6
Xbarc139
Xcfa2241
Xcfd4.2
Xbarc248
Xgwm566
Xgwm274.3
Xgwm375.1
Xgwm77
Xgwm285
Xgwm376.1
TC249615
Xgwm376.2
Xbarc203
Xbarc251
Xbarc164
XDuPw227
Xbarc115
qTN.LN-3B
qTN.CK-3B
qTN.LP-3B
qSDW.CK-3B
qSDW.LN-3B
qRDW.CK-3B
qNUP.CK-3B
qPUP.CK-3B
3D
Xbarc128.2
Xcfd4.1
Xbarc6
Xcfd70
Xbarc125
Xbarc1119
Xgwm456
Xcfd62
Xgdm136.1
Xbarc226
Xgwm645
Xcfd152.2
Xgwm314
Xcfd223
Xgwm3
Xcfd9
Xgwm114
4A
Xgwm601
Xbarc138
Xbarc106
Xbarc190
Xbarc237
Xgwm397
Xgwm383
Xbarc170
Xbarc1136.2
Xbarc236
Xbarc1158
Xbarc1047
Xbarc223.1
Xcfd20.2
Xac1.1
Xgwm160
Xbarc78
Xgwm350.1
Xbarc52
Xbarc70.1
Xbarc244
Xbarc153
Xcfd31
Xbarc246.1
4B
Xbarc193
TC246843
XDuPw270
Xgwm192.1
Xgwm368
Xbarc90
Xbarc20
Xgwm107.1
Xbarc199
TC233717
Xgwm165.1
Xgwm375.2
Xgwm251
XDuPw259
XDuPw619
Xgwm538
XDuPw33
XDuPw278
Xbarc114
qPUP.CK-4B
qSDW.LN-4B
qNUP.CK-4B
qNUP.LN-4B
qPUP.LN-4B
qRDW.CK-4B
qRDW.LN-4B
qSDW.CK-4B
qSDW.LP-4B
qRDW.LP-4B
qNUP.LP-4B
qPUP.LP-4B
4D
Xgdm14.5
Xgwm55.4
XDuPw238
Xbarc98
Xgwm55.2
Xgdm14.2
Xcfd193
Xcfd71
Xbarc105
Xgwm192.2
Xgwm165.2
TC237440
qNUP.LP-4D
qRDW.LN-4D
qPUP.LN-4D

## Slide 4
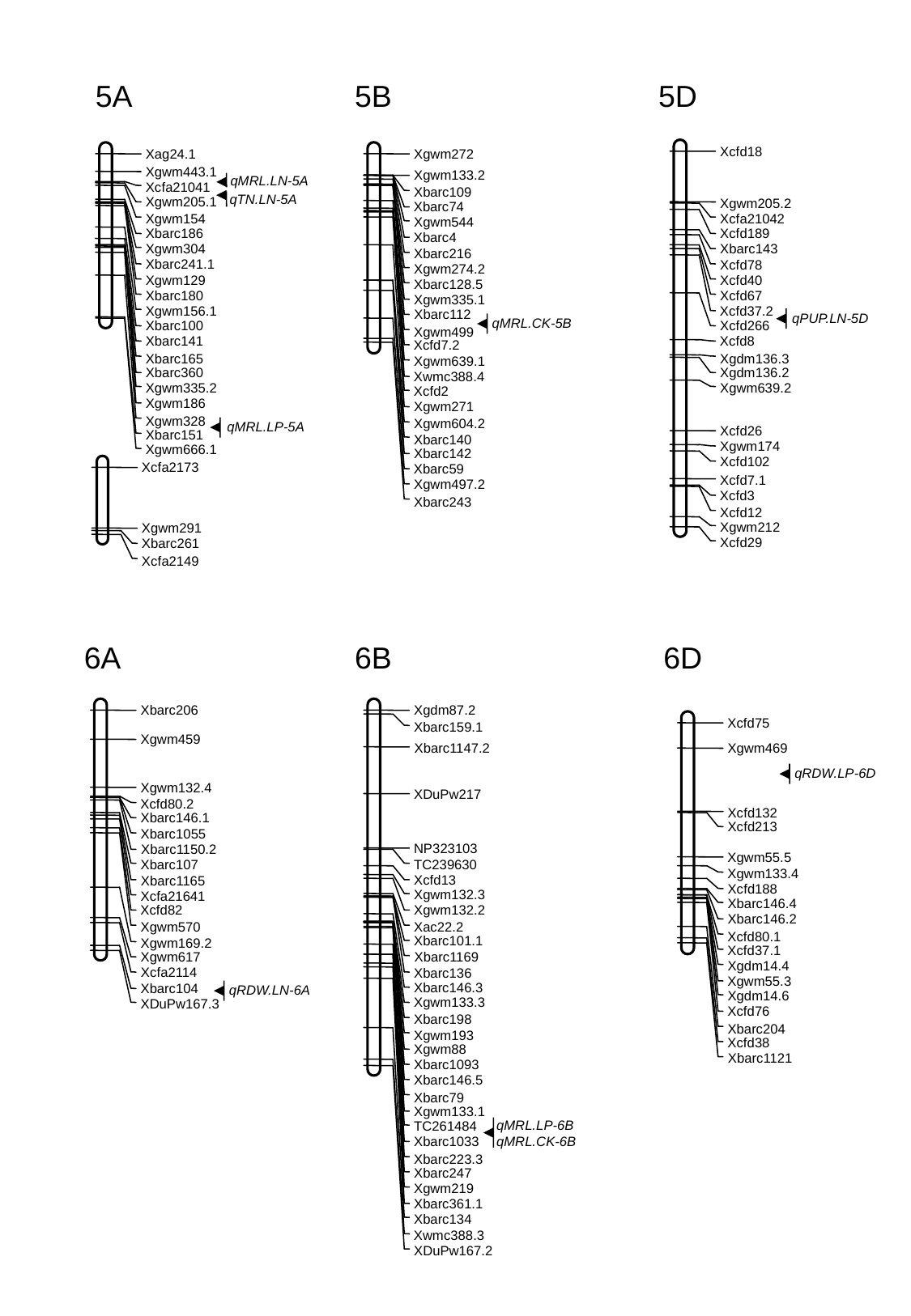

5A
Xag24.1
Xgwm443.1
qMRL.LN-5A
Xcfa21041
qTN.LN-5A
Xgwm205.1
Xgwm154
Xbarc186
Xgwm304
Xbarc241.1
Xgwm129
Xbarc180
Xgwm156.1
Xbarc100
Xbarc141
Xbarc165
Xbarc360
Xgwm335.2
Xgwm186
Xgwm328
qMRL.LP-5A
Xbarc151
Xgwm666.1
Xcfa2173
Xgwm291
Xbarc261
Xcfa2149
5B
Xgwm272
Xgwm133.2
Xbarc109
Xbarc74
Xgwm544
Xbarc4
Xbarc216
Xgwm274.2
Xbarc128.5
Xgwm335.1
Xbarc112
Xgwm499
Xcfd7.2
Xgwm639.1
Xwmc388.4
Xcfd2
Xgwm271
Xgwm604.2
Xbarc140
Xbarc142
Xbarc59
Xgwm497.2
Xbarc243
qMRL.CK-5B
5D
Xcfd18
Xgwm205.2
Xcfa21042
Xcfd189
Xbarc143
Xcfd78
Xcfd40
Xcfd67
Xcfd37.2
Xcfd266
Xcfd8
Xgdm136.3
Xgdm136.2
Xgwm639.2
Xcfd26
Xgwm174
Xcfd102
Xcfd7.1
Xcfd3
Xcfd12
Xgwm212
Xcfd29
qPUP.LN-5D
6A
Xbarc206
Xgwm459
Xgwm132.4
Xcfd80.2
Xbarc146.1
Xbarc1055
Xbarc1150.2
Xbarc107
Xbarc1165
Xcfa21641
Xcfd82
Xgwm570
Xgwm169.2
Xgwm617
Xcfa2114
Xbarc104
XDuPw167.3
qRDW.LN-6A
6B
Xgdm87.2
Xbarc159.1
Xbarc1147.2
XDuPw217
NP323103
TC239630
Xcfd13
Xgwm132.3
Xgwm132.2
Xac22.2
Xbarc101.1
Xbarc1169
Xbarc136
Xbarc146.3
Xgwm133.3
Xbarc198
Xgwm193
Xgwm88
Xbarc1093
Xbarc146.5
Xbarc79
Xgwm133.1
TC261484
Xbarc1033
Xbarc223.3
Xbarc247
Xgwm219
Xbarc361.1
Xbarc134
Xwmc388.3
XDuPw167.2
qMRL.LP-6B
qMRL.CK-6B
6D
Xcfd75
Xgwm469
Xcfd132
Xcfd213
Xgwm55.5
Xgwm133.4
Xcfd188
Xbarc146.4
Xbarc146.2
Xcfd80.1
Xcfd37.1
Xgdm14.4
Xgwm55.3
Xgdm14.6
Xcfd76
Xbarc204
Xcfd38
Xbarc1121
qRDW.LP-6D

## Slide 5
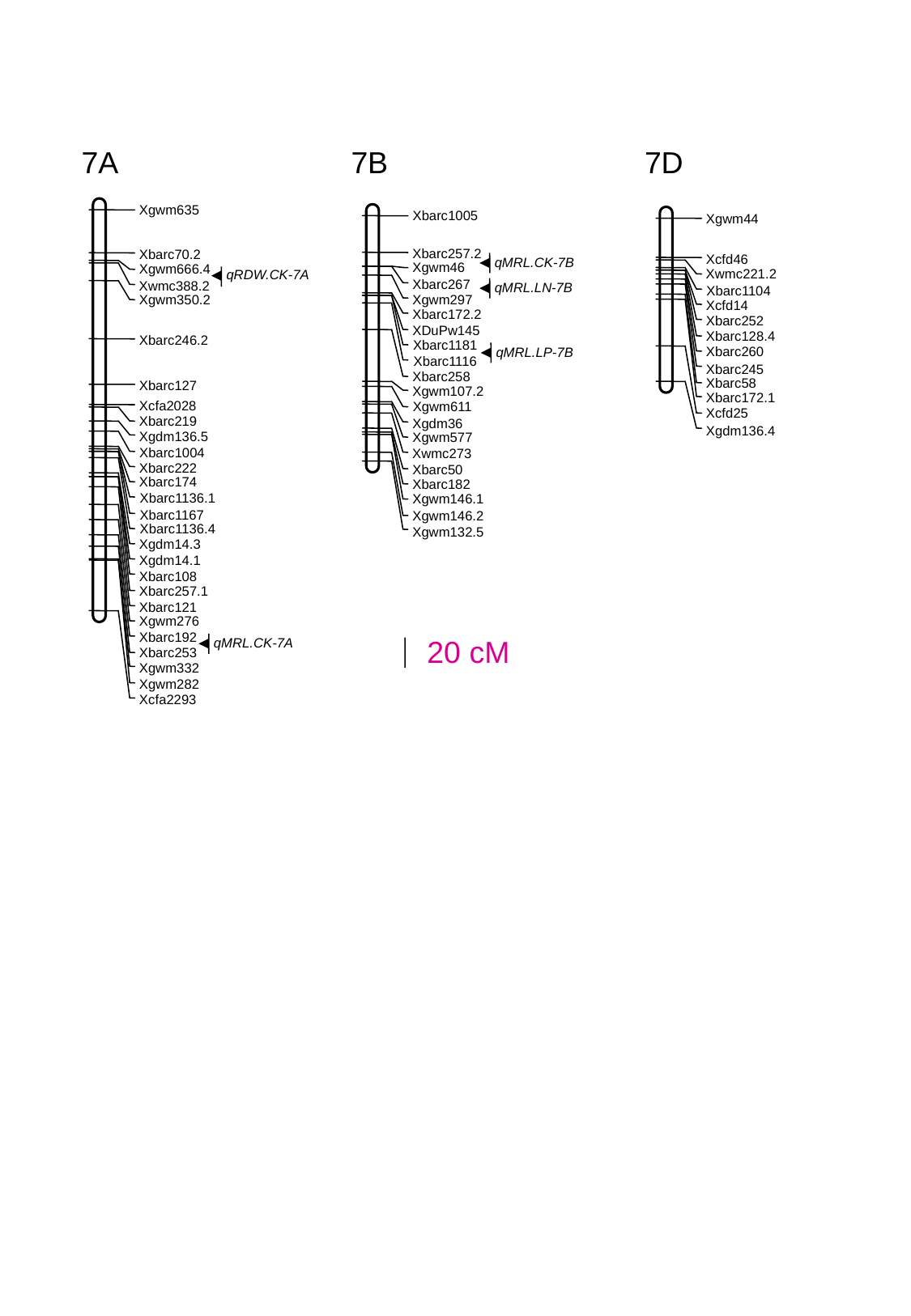

7A
Xgwm635
Xbarc70.2
Xgwm666.4
Xwmc388.2
Xgwm350.2
Xbarc246.2
Xbarc127
Xcfa2028
Xbarc219
Xgdm136.5
Xbarc1004
Xbarc222
Xbarc174
Xbarc1136.1
Xbarc1167
Xbarc1136.4
Xgdm14.3
Xgdm14.1
Xbarc108
Xbarc257.1
Xbarc121
Xgwm276
Xbarc192
Xbarc253
Xgwm332
Xgwm282
Xcfa2293
qRDW.CK-7A
qMRL.CK-7A
7B
Xbarc1005
Xbarc257.2
Xgwm46
Xbarc267
Xgwm297
Xbarc172.2
XDuPw145
Xbarc1181
Xbarc1116
Xbarc258
Xgwm107.2
Xgwm611
Xgdm36
Xgwm577
Xwmc273
Xbarc50
Xbarc182
Xgwm146.1
Xgwm146.2
Xgwm132.5
qMRL.CK-7B
qMRL.LN-7B
qMRL.LP-7B
7D
Xgwm44
Xcfd46
Xwmc221.2
Xbarc1104
Xcfd14
Xbarc252
Xbarc128.4
Xbarc260
Xbarc245
Xbarc58
Xbarc172.1
Xcfd25
Xgdm136.4
20 cM
