# Supplementary material for: Characterization of QTLs for Root Traits of Wheat Grown under Different Nitrogen and Phosphorus Supply Levels
Source: Front Plant Sci. 2017 Dec 11;8:2096. doi: 10.3389/fpls.2017.02096 (PMC5732362; doi:10.3389/fpls.2017.02096)
Supplement: Table S2 — Mean values and ranges for tiller number, shoot dry weight, nitrogen and phosphorus uptake in the RIL population and their parents under CK, low N, and low P conditions. [file Tables2-5.DOC]

**Table S2** Mean values and ranges for tiller number, shoot dry weight, nitrogen and phosphorus uptake in the RIL population and their parents under CK, low N and low P conditions

| Trait | Treat-  ment | Parent（Mean ± SE） | |  | RIL | | |
| --- | --- | --- | --- | --- | --- | --- | --- |
|  |  | Xiaoyan54 | Jing 411 |  | Mean ± SD | Min. | Max. |
| TN | CK | 3.3 ± 0.4 | 2.8 ± 0.2 |  | 3.1 ± 0.8 | 1.5 | 7.5 |
|  | Low N | 2.3 ± 0.4 | 1.7 ± 0.2 |  | 2.1 ± 0.6 | 3.3 | 1.0 |
|  | Low P | 1.8 ± 0.2 | 1.3 ± 0.2 |  | 1.8 ± 0.4 | 2.8 | 1.0 |
| SDW | CK | 0.330 ± 0.080 | 0.393 ± 0.060 |  | 0.367 ± 0.093 | 0.184 | 0.661 |
|  | Low N | 0.217 ± 0.030 | 0.231 ± 0.020 |  | 0.254 ± 0.056 | 0.137 | 0.433 |
|  | Low P | 0.267 ± 0.018 | 0.249 ± 0.012 |  | 0.261 ± 0.058 | 0.135 | 0.411 |
| NUP | CK | 17.1 ± 4.0 | 20.6 ± 3.3 |  | 19.4 ± 5.2 | 9.3 | 36.0 |
|  | Low N | 6.7 ± 0.7 | 6.2 ± 0.5 |  | 7.5 ± 1.5 | 4.5 | 12.1 |
|  | Low P | 11.7 ± 0.9 | 9.6 ± 0.3 |  | 10.9 ± 2.2 | 5.7 | 15.9 |
| PUP | CK | 3.80 ± 0.87 | 3.58 ± 0.58 |  | 3.88 ± 1.01 | 1.83 | 7.28 |
|  | Low N | 3.12 ± 0.34 | 2.10 ± 0.17 |  | 2.72 ± 0.58 | 1.57 | 4.60 |
|  | Low P | 0.59 ± 0.05 | 0.44 ± 0.01 |  | 0.59 ± 0.12 | 0.58 | 0.95 |

TN, tiller numbers per plant; SDW, shoot dry weight (g plant^-1^); NUP, N accumulated in the shoot per plant (mg plant^-1^); PUP, P accumulated in the shoot per plant (mg plant^-1^).

**Table S3** Correlation coefficients between the traits under CK, low N and low P conditions

| Treatment | Trait | TN | SDW | RDW | MRL | NUP |
| --- | --- | --- | --- | --- | --- | --- |
| CK | SDW | 0.525** |  |  |  |  |
|  | RDW | 0.473** | 0.868** |  |  |  |
|  | MRL | 0.156** | 0.240** | 0.307** |  |  |
|  | NUP | 0.550** | 0.989** | 0.893** | 0.230** |  |
|  | PUP | 0.534** | 0.931** | 0.877** | 0.214** | 0.937** |
| Low N | SDW | 0.425** |  |  |  |  |
|  | RDW | 0.364** | 0.809** |  |  |  |
|  | MRL | 0.185** | 0.241** | 0.430** |  |  |
|  | NUP | 0.422** | 0.947** | 0.872** | 0.325** |  |
|  | PUP | 0.390** | 0.841** | 0.826** | 0.255** | 0.888** |
| Low P | SDW | 0.490** |  |  |  |  |
|  | RDW | 0.425** | 0.790** |  |  |  |
|  | MRL | 0.127** | 0.091 | 0.330** |  |  |
|  | NUP | 0.498** | 0.949** | 0.860** | 0.139** |  |
|  | PUP | 0.459** | 0.815** | 0.787** | 0.184** | 0.874** |

** Correlation is significant at the 0.01 level (2-tailed).

**Table S4** Pyramiding QTLs for root biomass detected under low nitrogen condition enhanced N and P uptake

| Trait | Treatment | ***Xbarc90-4B + Xgwm165.2-4D + Xdwpw167.3-6A*** | | Increase (%) |
| --- | --- | --- | --- | --- |
|  |  | Positive (n=15) | Negative (n=10) |  |
| TN  (tiller/plant) | CK | 2.98 ± 0.45 | 2.97 ± 0.64 | 0.4 |
|  | LN | 2.01 ± 0.49 | 2.25 ± 0.61 | -10.6 |
|  | LP | 1.73 ± 0.50 | 1.63 ± 0.46 | 6.1 |
| SDW  (g/plant) | CK | 0.430 ± 0.119 (A) | 0.307 ± 0.074 (B) | 40.0 |
|  | LN | 0.301 ± 0.064 (A) | 0.222 ± 0.045 (B) | 35.2 |
|  | LP | 0.291 ± 0.059 (A) | 0.216 ± 0.042 (B) | 35.1 |
| RDW  (g/plant) | CK | 0.078 ± 0.024 (a) | 0.055 ± 0.013 (b) | 42.3 |
|  | LN | 0.132 ± 0.036 (A) | 0.090 ± 0.021 (B) | 46.7 |
|  | LP | 0.098 ± 0.023 (a) | 0.076 ± 0.012 (b) | 29.6 |
| MRL  (cm) | CK | 35.4 ± 9.6 | 38.1 ± 7.9 | -7.1 |
|  | LN | 40.2 ± 9.2 | 37.2 ± 8.4 | 8.3 |
|  | LP | 33.6 ± 9.8 | 37.0 ± 10.1 | -9.1 |
| NUP  (mg/plant) | CK | 22.2 ± 6.2 (a) | 16.4± 4.3 (b) | 35.7 |
|  | LN | 8.7 ± 1.5 (A) | 6.5 ± 1.0 (B) | 34.0 |
|  | LP | 12.0 ± 2.2 (A) | 9.0 ± 1.2 (B) | 34.5 |
| PUP  (mg/plant) | CK | 4.43 ± 1.18 (a) | 3.38 ± 0.82 (b) | 31.1 |
|  | LN | 3.26 ± 0.71 (A) | 2.48 ± 0.39 (B) | 31.6 |
|  | LP | 0.66 ± 0.13 (A) | 0.49 ± 0.08 (B) | 33.8 |

Statistical difference between the positively and the negatively pyramiding groups is indicated by different letters after the means. Capital and small letters designate significance at P < 0.01 and P < 0.05, respectively.

**Table S5** Detected QTLs for tiller number, shoot dry weight, nitrogen and phosphorus uptake

| Trait^a^ | QTL | Chr^b^ | Marker interval^c^ | LOD^d^ | *R*^2^  × 100 | Additive^e^ |
| --- | --- | --- | --- | --- | --- | --- |
| TN  (tiller/plant) | *qTN.CK-2A* | 2A | *Xgwm448-Xcfa2043* | 2.8 | 7.8 | -0.2 |
|  | *qTN.CK-3B* | 3B | *Xgwm533.1-Xbarc133* | 7.6 | 22.8 | 0.4 |
|  | *qTN.LN-1B* | 1B | *Xgwm403-Xbarc1129* | 2.5 | 6.5 | 0.1 |
|  | *qTN.LN-3B* | 3B | *Xbarc238-Xgwm533.1* | 7.3 | 22.6 | 0.3 |
|  | *qTN.LN-5A* | 5A | *Xcfa21041-Xgwm205.1* | 2.8 | 6.1 | -0.1 |
|  | *qTN.LP-1A* | 1A | *Xbarc120.1-Xgwm164* | 3.6 | 8.7 | -0.1 |
|  | *qTN.LP-2A* | 2A | *Xbarc1150.1-Xgwm448* | 4.3 | 9.6 | -0.1 |
|  | *qTN.LP-2D* | 2D | *Xgwm356-Xcfd50* | 2.7 | 10.1 | 0.1 |
|  | *qTN.LP-3B* | 3B | *Xgwm533.1-Xbarc133* | 6.6 | 17.4 | 0.2 |
| SDW  (g/plant) | *qSDW.CK-2A* | 2A | *Xgwm448-Xcfa2043* | 3.2 | 8.4 | -0.028 |
|  | *qSDW.CK-2D* | 2D | *Xgwm157-Xgwm102* | 3.3 | 16.1 | 0.037 |
|  | *qSDW.CK-3B* | 3B | *Xgwm533.2-Xbarc101.2* | 3.6 | 10.6 | -0.030 |
|  | *qSDW.CK-4B* | 4B | *Xbarc20-Xgwm107.1* | 5.8 | 13.4 | -0.035 |
|  | *qSDW.LN-2A* | 2A | *Xgwm448-Xcfa2043* | 2.8 | 6.8 | -0.016 |
|  | *qSDW.LN-2D* | 2D | *Xgwm157-Xgwm102* | 5.4 | 30.8 | 0.034 |
|  | *qSDW.LN-3B* | 3B | *Xgwm533.2-Xbarc101.2* | 3.2 | 8.7 | -0.018 |
|  | *qSDW.LN-4B* | 4B | *Xgwm368-Xbarc90* | 6.3 | 14.6 | -0.023 |
|  | *qSDW.LP-1D* | 1D | *Xdwpw7.1-Xdwpw7.3* | 2.6 | 6.0 | -0.015 |
|  | *qSDW.LP-2A* | 2A | *Xbarc212-Xbarc1138.1* | 2.5 | 7.0 | 0.016 |
|  | *qSDW.LP-2D1* | 2D | *Xgwm157-Xgwm102* | 2.8 | 7.8 | 0.016 |
|  | *qSDW.LP-2D2* | 2D | *Xcfd50-Xgwm311* | 2.7 | 10.0 | 0.018 |
|  | *qSDW.LP-4B* | 4B | *Xbarc20-Xgwm107.1* | 7.5 | 16.5 | -0.025 |
| NUP  (mg/plant) | *qNUP.CK-3B* | 3B | *Xgwm533.2-Xbarc101.2* | 3.9 | 12.4 | -1.8 |
|  | *qNUP.CK-4B* | 4B | *Xgwm368-Xbarc90* | 4.3 | 10.8 | -1.7 |
|  | *qNUP.LN-2D* | 2D | *Xgwm539-Xgwm157* | 4.0 | 11.4 | 0.5 |
|  | *qNUP.LN-4B* | 4B | *Xgwm368-Xbarc90* | 6.3 | 15.0 | -0.6 |
|  | *qNUP.LP-2A* | 2A | *Xbarc212-Xbarc1138.1* | 2.6 | 8.3 | 0.7 |
|  | *qNUP.LP-2D* | 2D | *Xcfd50-Xgwm311* | 2.8 | 9.0 | 0.7 |
|  | *qNUP.LP-4B* | 4B | *Xgwm165.1-Xgwm375.2* | 4.2 | 10.1 | -0.7 |
|  | *qNUP.LP-4D* | 4D | *Xgwm192.2-Xgwm165.2* | 2.9 | 7.5 | 0.6 |
| PUP  (mg/plant) | *qPUP.CK-2D* | 2D | *Xgwm157-Xgwm102* | 3.4 | 12.3 | 0.36 |
|  | *qPUP.CK-3B* | 3B | *Xgwm533.2-Xbarc101.2* | 3.2 | 10.5 | -0.33 |
|  | *qPUP.CK-4B* | 4B | *Xbarc193-T6843* | 3.6 | 13.3 | -0.37 |
|  | *qPUP.LN-4B* | 4B | *Xgwm368-Xbarc90* | 5.7 | 14.3 | -0.24 |
|  | *qPUP.LN-4D* | 4D | *Xgwm165.2-NP_39* | 4.7 | 13.6 | 0.24 |
|  | *qPUP.LN-5D* | 5D | *Xcfd37.2-Xcfd266* | 3.0 | 11.1 | -0.20 |
|  | *qPUP.LP-2A* | 2A | *Xbarc1138.1-Xgwm614.2* | 3.2 | 13.4 | 0.05 |
|  | *qPUP.LP-2D* | 2D | *Xcfd50-Xgwm311* | 3.2 | 18.9 | 0.05 |
|  | *qPUP.LP-4B* | 4B | *Xgwm165.1-Xgwm375.2* | 6.6 | 16.7 | -0.05 |

^a^ TN, tiller number per plant; SDW, shoot dry weight; NUP, nitrogen uptake; PUP, phosphorus uptake.

^b^ Chr means chromosome name.

^c^ Markers underlined were the nearest marker to the QTL.

^d^ LOD means Logarithm of odds.

^e^ Additive effects, a positive sign means that positive allele comes from the parent Xiaoyan 54, while a negative sign means positive allele comes from the parent Jing 411.
